# PASS Theory and Movement Disorders: Methodology for Assessment and Intervention

**DOI:** 10.3390/children11101192

**Published:** 2024-09-29

**Authors:** Katerina Asonitou, Dimitra Koutsouki

**Affiliations:** Laboratory of Adapted Physical Activity/Developmental and Physical Disabilities, School of Physical Education and Sport Science, National and Kapodistrian University of Athens, 17237 Athens, Greece; dkoutsou@phed.uoa.gr

**Keywords:** planning, executive functions, cognitive-motor assessment, comorbidity, DCD, early intervention, preschool age

## Abstract

Background/Objectives: Executive dysfunction appears to be a significant secondary characteristic frequently linked with movement disorders. Planning is a high-level cognitive process integral to our executive functions. Children who show deficits in their planning ability usually have difficulties in making decisions or problem-solving, such as initiating tasks or mentally organizing a project, and monitoring and controlling their behavior effectively. These difficulties can significantly impact their academic performance and daily functioning even in adulthood. However, the existing research on the relationships between motor performance and planning abilities is limited and inconsistent. Methods: This study aimed to explore the potential associations between motor and planning skills in 30 preschool-aged children with Developmental Coordination Disorder (DCD) and 30 healthy controls matched for age, including utilizing the PASS theory and Cognitive Assessment System (CAS). Results: The findings highlighted balance, total motor score, and planned codes as the primary factors distinguishing the two groups. A significant Pearson’s correlation was observed between the overall MABC-2 scores and the subdomain scores, along with the Planning Scale indices for both groups, suggesting a substantial relationship between these assessments. Among the Planned codes items, the most notable predictor of overall motor performance in children was identified. Furthermore, the total score for Planned Connections emerged as the most robust predictor for tasks associated with manual dexterity. Conclusions: The relationship between motor skills and executive functions in early childhood plays a vital role in the development of early intervention strategies that utilize cognitive-motor tools.

## 1. Introduction

Cognitive and motor development are fundamentally interconnected, as highlighted in the literature. It is crucial to identify delays in both cognitive and motor skills across typically and atypically developing populations. This examination helps to understand the degree to which these domains may diverge during a child’s development, which is vital for clinical assessments and educational interventions, particularly concerning neurodevelopmental disorders such as ASD, ID, DCD, ADHD, and dyslexia. These disorders have a neurobiological foundation and typically present in early childhood. While some children may overcome their motor challenges with or without support, others continue to experience movement difficulties into adolescence and adulthood [1,2,3].

Motor skills are supported by cognitive processes referred to as executive functions (EFs), which are responsible for regulating, monitoring, and controlling behavior in pursuit of a goal. EFs serve as a significant predictor of academic success during childhood and maintain their predictive power for overall life success in adulthood. Understanding executive functions is crucial for improving life outcomes for children with motor difficulties [4,5]. This literature review identifies deficits in both fine and gross motor skills, along with executive functions (EFs)—which include planning, task switching, response inhibition, and memory storage—as two prominent characteristics of DCD [6,7,8]. Developmental Coordination Disorder (DCD) is a significant challenge in the development and execution of age-appropriate motor skills, which can severely impact children’s daily activities and academic performance. This condition is not associated with intellectual disabilities or neurological disorders that affect movement, such as cerebral palsy or muscular dystrophy [9]. Children with DCD often struggle to identify similarities between different motor tasks, which hinders their ability to transfer motor skills from one activity to another. For instance, they may find it difficult to transition from catching a large ball to catching a smaller one, or they may not recognize that stepping up onto a sidewalk is similar to climbing stairs. Additionally, they face challenges in adapting to changing environments, as they have difficulty processing new information and responding appropriately in a timely manner, such as when trying to catch or hit a moving ball or avoiding collisions during team sports. The consequence of any of the aforementioned issues is their challenge in acquiring and executing new motor skills. The challenges encountered by children extend beyond mere motor difficulties, highlighting the social and emotional impacts of this disorder, particularly the feeling of exclusion during playtime. Their coping strategies reflect their attempts to foster resilience [10].

Current estimates indicate that the prevalence of DCD among children varies between 2% and 20% [11], with a commonly accepted prevalence rate in the literature of 5–6% [9]. The global prevalence rates among school-aged children underscore the widespread occurrence of this neurodevelopmental disorder. It is well-recognized that children with DCD face considerable challenges in motor skills, which can hinder their social interactions and academic achievements. The disorder can have a profound impact on multiple developmental domains, including cognitive and social-emotional development [12], as well as overall quality of life [10].

**Figure 1:**
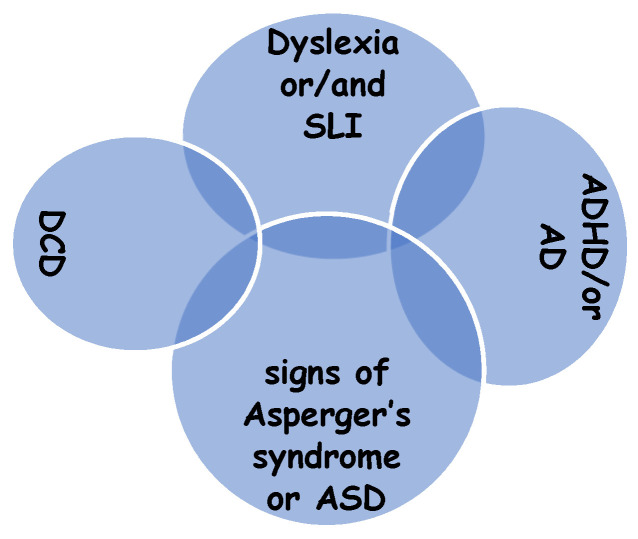
Comorbidity of DCD [16].

Students diagnosed with Developmental Coordination Disorder (DCD) represent a diverse group marked by challenges in acquiring or automating motor skills. The variability in executive functions (EFs) among co-occurring neurodevelopmental disorders, such as Autism Spectrum Disorder (ASD) and Attention-Deficit/Hyperactivity Disorder (ADHD), presents specific opportunities for targeted EF interventions [13]. Additionally, the prevalence of comorbidity is notably high among children with generalized perceptual-motor disorders [9,14]. DCD is frequently linked with attention hyperactivity disorders (35–50%), specific language impairments (SLI), dyslexia (35%), dyscalculia, ASD, Asperger’s Syndrome, and Tourette’s Syndrome, as well as anxiety, depression, and difficulties in social interaction and self-esteem (Figure 1). In clinical populations with DCD, comorbidity is more often the norm than the exception [15,16].

**Figure 2:**
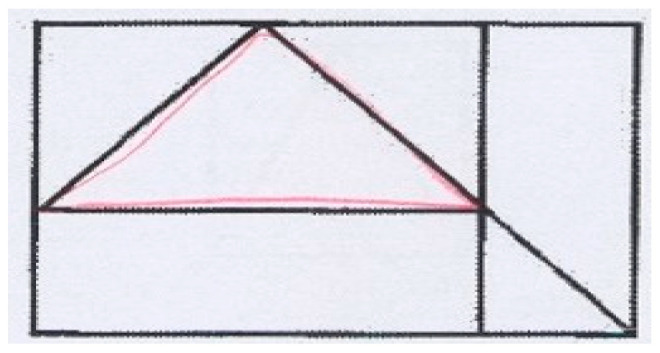
Embedded schema derived from the figure memory item of the CAS.

The neuropsychological model of cognitive functions proposed by Luria [17] (1966) outlines three fundamental characteristics of information processing for any activity. The process involves three main steps: 1. re-coding information; 2. organizing action plans and choosing appropriate responses; and 3. assessing the results of the actions against the original goals. Motor development is linked to other areas of growth, like cognitive and emotional development, and is a strong indicator of future cognitive skills. All motor activities are connected to cognitive functions. Internal speech is vital for planning motor tasks, serving as a quiet link between thought and action [18]. A clear example of motor planning is the task of drawing a shape, like a cube, which requires accuracy and skill in both encoding and processing [Figure 2]. The child first needs to sketch the shape from memory, which involves processing at the same time, and then build the shape through a series of steps, requiring processing in sequence.

**Figure 3:**
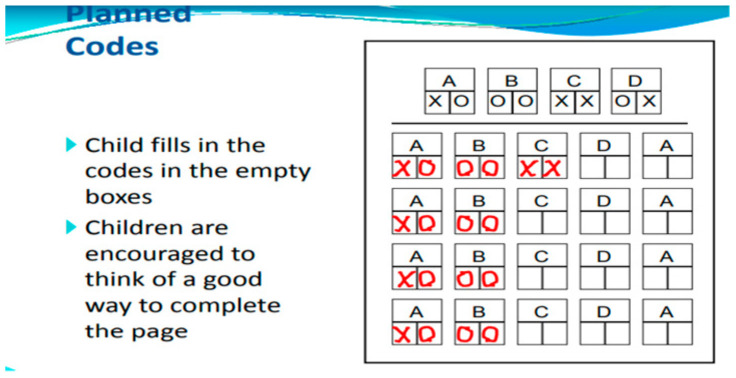
Planned Codes subtest1. Children fill in the codes in the blank boxes. They are encouraged to come up with a creative way to finish the page.

**Figure 4:**
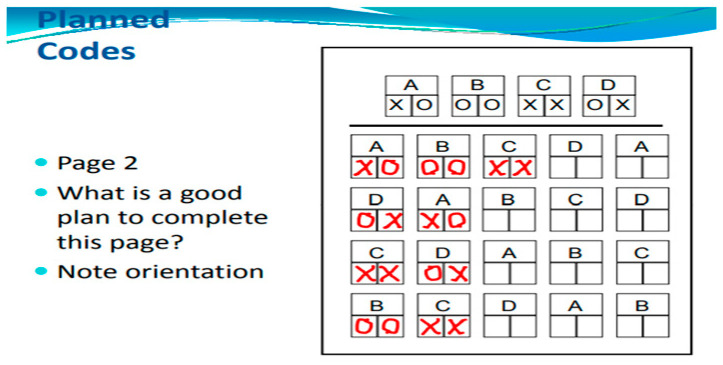
Planned Codes subtest2. What is a good plan to complete this page? Note orientation.

Planning is a way of thinking about how to accomplish a task. The Planned Codes subtest serves as an excellent illustration of a task that can be approached with a strategic method. In this subtest, children are tasked with writing a designated letter code beneath the appropriate letter (for instance, XO for A, OX for B, and so forth). Many children adopt a strategy of first completing all the As followed by the Bs, leading to improved scores compared to those who do not employ such a strategy (Figure 3 and Figure 4).

PASS processes are linked to specific brain structures, indicating that they support a range of processes that contribute to various cognitive and motor behaviors. The integration of PASS theory with Luria’s neuropsychological model establishes a relationship between motor skills and cognitive processes [19,20]. The PASS theory and the Cognitive Assessment System (CAS) can be utilized to pinpoint dysfunctions in information processing capabilities, specifically in the areas of (a) planning and motor programming, (b) failures in coding, whether simultaneous or successive, (c) the evaluation of feedback or the inability to utilize feedback when it is available, and (d) the presence of an adequate knowledge base. Consequently, children may enhance their skills in specific areas, such as motor control, pattern following, error recognition, and the application of strategies, based on advice received. Das (1986) [21] posits that the comprehension of movement encompasses two primary aspects: (1) the significance of effective planning and decision-making in achieving proficiency in motor performance, and (2) consequently, the interrelation between the ‘motor’ and ‘mental’ domains, indicating that they are not separate entities. Inappropriate levels of attention or arousal can interfere with planning, thereby impacting working memory and processing speed, which in turn affects various achievement domains. Even when attention or arousal remains within acceptable limits, difficulties in planning may still hinder lower levels of cognitive processing. Additionally, weaknesses in one or more general cognitive skills can lead to specific learning challenges across different achievement areas, particularly resulting in notable deficits in processing speed. For instance, inadequate information processing may hinder word decoding, leading to an overreliance on visual cues in spelling and difficulties in adhering to a problem-solving strategy. Ultimately, these learning challenges can also lead to secondary emotional issues, which may further influence higher-level cognitive processing [22].

The integration of the PASS theory and CAS as a cognitive assessment tool for motor development offers a comprehensive conceptual framework, facilitating a more holistic approach to the assessment, prescription, and intervention strategies for children experiencing movement challenges [18]. Our earlier research explored the characteristics of potential cognitive-motor profiles associated with and without Developmental Coordination Disorder (DCD) through clustering techniques, identifying six distinct cognitive-motor profiles. Each group within the DCD classification exhibited varying levels of severity of the disorder [15].

Recognizing developmental delays in cognitive and motor skills, as well as exploring how these areas may diverge during a child’s growth, is crucial for clinical evaluation and educational support. The prompt detection of cognitive and motor challenges may serve as an indicator of future academic success and plays a vital role in facilitating early intervention.

## 2. Aim of the Research

The objectives of the research were twofold: (a) to evaluate and contrast the motor performance and planning capabilities of children diagnosed with Developmental Coordination Disorder (DCD) against those without the condition, utilizing the PASS theory and Cognitive Assessment System (CAS), and (b) to assess the role of cognitive planning in influencing their motor performance.

## 3. Target Group

Ν = 60 students: (n1 = 30) with DCD (n2 = 30) age-matched with typical development / 38 boys and 22 girls. 5-year-old, mean age = 61.84 months, (range 55–65), 6-year-old, mean age = 69.40 months, (range 66–76). They were attending public kindergartens and had no pediatric histories of any physical or neuromotor disorder (CP or MD), ADHD (attention-deficit /hyperactivity disorder), or emotional or intellectual disability (i.e., no children had IQ less than 80).

- Sample size with power analysis: 0.64 effect size (95% CI = 0.44–0.78) [23]. Minimum sample: Ν = 36 students.
- Students: age matched. Purposeful sampling [24].

## 4. Methods and Measures

The working method involved the collection of data on children’s cognitive abilities and motor development skills through standardized assessments. The broad categorization into groups may seem overly simplistic in certain respects; however, evaluating various clinical groups provides a more accurate representation than merely contrasting a DCD group with a typically developing group. Adopting a multigroup and comorbidity-focused strategy has been proposed as a crucial measure for recognizing the distinct motor performance variations associated with each developmental disorder [14].

The DSM-5, published by the American Psychiatric Association in 2013, serves as the current framework for diagnosing Developmental Coordination Disorder (DCD). It outlines four specific criteria for this diagnosis. Criterion A states that the development and execution of coordinated motor skills are significantly below what is expected for an individual’s chronological age and their opportunities for skill acquisition. Criterion B indicates that the motor skill deficits identified in Criterion A substantially and consistently hinder daily living activities, affecting productivity as well as prevocational and vocational tasks, along with leisure and play. Criterion C specifies that the symptoms must manifest during the early developmental period. Finally, Criterion D clarifies that the motor skill deficits cannot be better accounted for by intellectual disabilities, visual impairments, or any neurological conditions [9]. So, it is significant to compare subjects with DCD with subjects with typical development. 

### 4.1. Independent Variables

The independent variables utilized in this research are presented in Table 1, followed by a discussion of the dependent variables. 

### 4.2. Dependent Variables

(a) The Planning Scale Scores from the Cognitive Assessment System, which includes three cognitive components: Number Matching, Planned Coding, and Planned Connections. 

(b) The Scores from the Movement Assessment Battery for Children-2 across three motor domains: Manual Dexterity, Aiming and Catching, and Static and Dynamic Balance.

- Scores of 5% or lower indicate “definite motor problems”.
- Scores between 6% and 15% suggest “borderline—at risk”.
- Scores exceeding 15% reflect “no motor difficulties”.

This was an initial study and all essential ethical research principles were adhered to: authorization was obtained from the Ministry of Education, communication was established with the teachers, and a leaflet outlining DCD characteristics was provided to assist teachers in the identification process. Informed consent forms were collected from the parents for every child involved. Each child participated in nine motor tasks from the MABC-2 and seven cognitive tasks from the CAS. The assessments of the students were conducted in the classroom, ensuring ecological validity. 

### 4.3. Measuring Instruments

i. Movement Assessment Battery for Children-2, (MABC-2). Ref. [25] (5:0–7:11 age band).

MABC-2 was utilized to assess the motor performance of children. The motor tasks are categorized into three main groups: (1) manual dexterity, which includes activities such as posting coins, threading beads, and navigating a bicycle trail; (2) aiming and catching, featuring tasks like catching a beanbag and throwing it towards a target; and (3) balance, encompassing activities such as balancing on one leg, walking with heels elevated, and jumping on mats. For this research, the first age band of the instrument was selected. The raw data were documented according to a time scale, as well as the count of errors and successful hits, and the motor task scores were standardized.

ii. The Planning Scale of the Cognitive Assessment System, (CAS). Ref. [26] (3:0–6:11 age band).

Planning Scale. Planning is a cognitive function linked to executive skills, involving the identification, selection, and execution of effective solutions. The Planning scale includes tasks such as matching numbers, planned codes, and planned connections. (i) In the matching numbers subtest, children are presented with four pages, each containing eight rows of numbers, where they must underline pairs of identical numbers. (ii) The planned codes subtest features two pages, each with a distinct set of codes arranged in seven rows and eight columns. A key at the top of each page indicates the relationship between letters and simple codes (for example, A = OX; B = XX; C = OO). (iii) The planned connections subtest, a variation of the Trails test, requires the child to connect numbers in a sequential order from a semi-random layout (for instance, 1–2–3, etc.). In these two assessments, the child alternates between connecting numbers and letters in the correct sequence (for example, 1-A-2-B, etc.). The outcomes provide standardized scores across seven cognitive domains based on the time taken and the number of correct answers.

## 5. Results

Statistical Analysis used SPSS 0.28, * Significance: 0.05.

Descriptive frequency analysis was used to obtain the actual percentages that corresponded below or above the criteria set by MABC-2′s and Planning Scale’s norms (Table 2 and Table 3). Multivariate analysis of variance (MANOVA) was used to examine the possible differences between the 2 groups in the 9 motor and 7 cognitive domains. Discriminant function analysis was used as a post hoc analysis indicating which items significantly separated the two groups. 

Stepwise multiple regression was used to predict children’s motor performance by using indicators from planning ability. In the current study, students with DCD had significantly lower scores than students without DCD in all three motor domains and the total motor score (MABC-2) as well as in all Planning scale scores, which is in accordance with relevant references in the literature [27,28,29,30]. Balance, total motor score and planned codes appeared to be the best discriminators between the two groups, indicating the strengths and weaknesses for both groups in specific skills.

We examined the differences between children with and without DCD, in the mean vector of scores with the following three Planning cognitive tasks: Matching numbers, Planned codes, and Planned connections. The results of the multivariate analysis of variance (MANOVA) in children with and without DCD aged 5–6 supported the existence of statistically significant differences between two groups in the linear combination of the 16 dependent cognitive-motor variables of the MABC-2 and CAS. The discriminant analysis (as a post hoc analysis) classified 98.3% of the cases correctly with balance, total motor score and planned codes as the best discriminators to explain the differences between two groups. The results are presented in Table 4 and Table 5, respectively. 

The Pearson Correlation coefficient between total MABC-2 and domain subscores and the Planning Scale indices were statistically significant for the two groups, indicating the degree of association between the two tests. The results are presented in Table 6.

Multiple regression analyses were then performed to identify the significant determinants of the total impairment score (TIS) on the MABC-2 among the children with DCD. The age, sex, and physical activity level were forced into the regression model using the Stepwise method. After accounting for age, sex, and physical activity level, the Planning index remained significantly associated with the MABC-2 total impairment score (TIS) and explained 35.7% of the variance in children with DCD (*p* = 0.000). The Planned codes items seem to be the best predictor of Total Motor performance. The results are presented in Table 7.

Also, the total score of Planned Connections items seems to be the best predictor for the manual dexterity tasks. The results are presented in Table 8. 

## 6. Discussion

The findings indicate that inadequate planning skills are notably linked to a lack of visual-motor integration. A lower level of planning appears to have a significant correlation with motor performance in children with Developmental Coordination Disorder (DCD), increasing the likelihood of suboptimal motor development. It is feasible to predict children’s motor performance, particularly manual dexterity, by utilizing indicators derived from cognitive planning factors. Planning has been integrated into the broader category of Executive Functions (EF), which also includes inhibition and working memory [31]. The influence of cognitive ability on academic performance and the moderating impact of planning have been examined [32]. Planning is associated with academic performance, such as math computation problems [20], reading, reading comprehension (vocabulary and speed), writing, and recall memory of literature passages [33,34,35]. Also, various studies have indicated that intricate planning tasks, such as the Tower of London and Wisconsin Card Sorting, can serve as predictors for reading [36] and mathematics performance [37].

DCD is associated with problems in planning abilities, and children with DCD are at significant risk of school failure in different academic areas. The ability of information processing has a significant role in motor and cognitive learning. Planning and maintaining attention are cognitive processes that have important roles in the achievement of motor reaction and furthermore in the formation of motor patterns [6,38].

Research indicates that motor and cognitive skills develop along a parallel path [32,39]. Piaget was the first to explore the interrelationship between children’s cognitive and motor development, suggesting that intelligence evolves through interactions with the external environment [40]. Mobility plays a vital role in cognitive processes, facilitating the development of neural connections and cortico-differentiation [41]. As infants engage with their surroundings, their brains respond to this sensory input. Neuroimaging studies have demonstrated that increased physical activity enhances the growth of gray matter in the brain [39].

□ Motor and cognitive activities are developed through active engagement with the knowledge base and the three cognitive function systems: attention, coding, and planning.
□ The Das–Naglieri Cognitive Assessment System (CAS) applies the PASS theory to evaluate cognitive strengths and weaknesses, which can inform the creation of tailored instructional programs [42].
□ Timely identification of particular cognitive-motor challenges may serve as an indicator of academic performance and plays a crucial role in facilitating early intervention [6,16,43].
□ Improving the monitoring and evaluation processes in early childhood education necessitates the systematic observation and documentation of a child’s daily routine activities, which include reading, writing, play, learning, verbal communication, and physical movement.

Educational intervention practices and task-specific teaching methods enhancing cognitive abilities such as planning, decision- making processing, problem solving, evaluation, spatial organization, memory, and attention mechanisms in combination with motor skills may facilitate learning and academic performance. Why this intervention and not a different one? Probably because it has greater long-term maintenance of their cognitive and motor goals and acquired strategies than other types of intervention [44,45]. For instance, the instructional approach known as Planning Facilitation has been shown to assist students with learning disabilities and ADHD in enhancing their planning strategies while working on math worksheets, resulting in improved performance on norm-referenced assessments of mathematical achievement. Furthermore, these students demonstrated the ability to apply the strategies they learned to different mathematical contexts, indicating a significant transfer of skills. This method intentionally refrains from directly teaching strategies, as research suggests that the transfer of learning is more effectively accomplished through inductive reasoning rather than deductive reasoning [33].

Also, in the study by [46], an intervention program focused on cognitive development, known as the PASS Reading Enhancement Program (PREP), was executed as an online computer-assisted intervention (e-PREP) aimed at a group of young learners facing challenges in reading. This program incorporated a game-based cognitive intervention tool tailored for this age group. The results of the intervention were examined, demonstrating its success in improving reading abilities among those who were struggling.

Limitations of the study. The methodology employed in the current study for diagnosing Developmental Coordination Disorder (DCD) adhered closely to the criteria outlined in the DSM-5 (2013) [9]; however, several significant limitations became apparent upon completion of the research. The children identified with DCD exhibited a range of motor dysfunctions, categorized as either “at risk” or severe, and these dysfunctions varied in nature. This observation underscores the diversity among children with DCD. Furthermore, the diagnostic protocol for DCD was not implemented by an occupational health service, such as a pediatrician or therapist; instead, the researchers relied solely on the MABC-2. Furthermore, there was a lack of oversight regarding extracurricular activities, enabling both participants with and without DCD to affect their performance on the evaluated motor and cognitive assessments. Also, the Planning Scale, like any assessment tool, has both advantages and disadvantages that should be thoughtfully evaluated when using and interpreting it.

## 7. Conclusions

The significance of cognitive planning in the development of children’s motor skills is paramount, as it influences their capacity to strategize, arrange, and perform motor tasks. Cognitive planning encompasses various processes, including attention, memory, decision-making, and problem-solving, all of which are vital for the advancement and implementation of intricate motor skills.

- Movement Planning: Children use cognitive planning to design their movements before executing them. This includes assessing space, understanding the requirements of the activity, and predicting outcomes.
- Organization and Coordination: Cognitive planning helps children organize their movements in a way that is efficient and coordinated. This is particularly important in activities that require a combination of different motor skills.
- Perception and Adaptation: As children perform motor activities, cognitive planning allows them to monitor their performance and adjust their movements according to the conditions and demands of the activity.
- Development of Strategies: Through cognitive planning, children develop strategies for learning new motor skills, such as repetition and feedback from parents or instructors.

Overall, cognitive planning is fundamental for the development of motor skills, as it enables children to perform complex movements with greater accuracy and confidence. The Planning and problem-solving approach, recognized as cognitive strategy instruction, has been shown to be especially advantageous for students who need it the most, particularly those with low Planning scores [33]. Future developmental research is encouraged to incorporate cognitive strategy tools to analyze the unique characteristics of diverse populations, particularly those with DCD. Investigating the effects of DCD classification is essential and could extend to other developmental disorders. Understanding the implications of various DCD profiles may lead to significant advantages in implementing alternative and effective teaching methods, as well as early intervention programs, aimed at preventing motor learning disabilities and enhancing academic performance. Also, physical education (PE) teachers, trainers, educators, and therapists will be able to adapt their support, accordingly, implementing an Individualized Education Program (IEP) regardless of the diagnostic medical label a child receives.

## Figures and Tables

**Table 1:** Independent variables used in this study.

Independent Variables		
i. Group - 30 students with DCD - 30 students without DCD	ii. Gender - 38 boys - 22 girls	iii. Age - 5:0–5:11 yrs old - 6:0–6:11 yrs old

**Table 2:** Means and SDs of Standard score differences for DCD and non-DCD Groups in motor skills.

Mοtοr Variables	DCD		Non-DCD	
	M	SD	M	SD
Manual dexterity 1	1.65	1.29	0.38	0.7
Manual dexterity 2	3.36	1.24	1.06	1.25
Manual dexterity 3	2.53	1.92	0.53	0.73
Aiming and Catching 1	2.7	1.8	0.5	0.68
Aiming and Catching 2	2.73	1.4	0.47	0.86
Static Balance	2.2	1.57	0.48	0.79
Dynamic Balance 1	2.56	2.3	0.73	1.25
Dynamic Balance 2	2.46	1.67	0.75	1.2
Total MABC score	20.19	7.67	4.9	2.96

**Table 3:** Means and SDs of Standard score differences for DCD and non-DCD Groups in Planning Scale and Cognitive abilities.

Cognitive Variables	DCD		Non-DCD	
	M	SD	M	SD
Matching Numbers 1 (0–151 s)	130.3	33.1	85.5	32.42
Matching Numbers 1 (corrects)	6.76	1.57	7.9	0.57
Matching Numbers 2 (0–151 s)	150.3	3.65	148.6	13.14
Matching Numbers 2 (corrects)	3.03	1.60	4.66	1.26
Planned Codes 1 and 2 (0–121 s)	121.0	0.0	121.0	0.0
Planned Codes 1 (corrects)	10.8	6.18	19.5	7.86
Planned Codes 2 (corrects)	9.56	3.82	15.16	5.34
Planned Connections	5.67	3.75	10.13	2.5
Planning standard scaled total score	82.86	14.5	104.9	13.5

**Table 4:** A Multivariate Analysis of Variance (MANOVA) was conducted to assess the significance of mean differences between children diagnosed with and without DCD. MANOVA was significant. Note. * *p* < 0.05.

Effect	Wilks’s Lambda	*F*	Hypo *df*	Error *df*	*p*	Eta Squared
DCD/non DCD	0.014	211.159	14.00	42.00	0.000	0.986

**Table 5:** Discriminant Function Analysis to examine the motor and cognitive skills that separate students and the percentage of students who were classified.

Variable	Wilks’s Lambda	*p*	Unstand. Coeff.	Stand. Coeff.	Struct. Coeff.
DCD/non DCD					
Balance	0.358	0.000	−0.188	−0.590	0.531
Total MABC-2 score	0.299	0.000	0.237 Constant–1.209	1.378	0.832
Planned Codes	0.279	0.000	−0.089	−0.438	−380
98.3% of original grouped cases correctly classified					

**Table 6:** Pearson Correlation coefficient between total MABC-2 scores, domain subscores, and planning scale scores.

Coefficients Variables		Matching Numbers Anagogy Index	Planned Codes Anagogy Index	Planned Connections Anagogy Index	Plannning PASS Standard Scaled Score Anagogy Index
Pearson correlation	man.dex	−0.521 **	−0.466 **	−0.570 **	−0.586 **
	ball.sk	−0.384 **	−0.404 **	−0.441 **	−0.466 **
	balance	−0.446 **	−0.376 **	−0.525 **	−0.511 **
	tot.abc-2	−0.549 **	−0.508 **	−0.614 **	−0.629 **
	man.dex	60	60	60	60

**: *p* < 0.01.

**Table 7:** Results of multiple regression equations demonstrating the prediction of children’s motor performance from Planned Codes scores (cognitive skill).

Coefficients						
Model		Unstandardized Coefficients		Standardized Coefficients	t	Sig.
		B	Std. Error	Beta		
1	(Constant)	26.807	4.921		5.448	0.000
	Planned codes total standardized score	−2.369	0.802	−0.740	−2.953	0.000
a. Dependent Variable: Total MABC-2 score (TIS)						

R-square = 0.357, meaning that 35.7% of confidence interval could be explained by Planned Codes items. Υ = 26.807 − 2.369 X _Planned codes_ − 1.370X_Matching numbers_ − 2.706X_Planning scale_ − 2.319X_Planned connections._

**Table 8:** Results of multiple regression equations regarding prediction of children’s motor performance from scores at Planned Connections Items (cognitive skills).

Coefficients						
Model		Unstandardized Coefficients		Standardized Coefficients	t	Sig.
		B	Std. Error	Beta		
1	(Constant)	9.311	0.964		9.655	−0.000
	Planned connections total standardized score	–0.311	−0.143	−0.381	−2.179	−0.003
a. Dependent Variable: manual dexterity						

R-square= 0.270 meaning that 27.0% of confidence interval could be explained by planned connections items. Υ= 9.311 − 0.311 X_planned connections_ + 0.105X_plannedcodes1_ + 0.131X_plannedcodes2_ + 0.232X_matching numbers._

## Data Availability

The data cannot be shared because the initial informed consent ensured privacy.
