# Correction: Asonitou, K.; Koutsouki, D. PASS Theory and Movement Disorders: Methodology for Assessment and Intervention. *Children* 2024, *11*, 1192

**DOI:** 10.3390/children11111406

**Published:** 2024-11-20

**Authors:** Katerina Asonitou, Dimitra Koutsouki

**Affiliations:** Laboratory of Adapted Physical Activity/Developmental and Physical Disabilities, School of Physical Education and Sport Science, National and Kapodistrian University of Athens, 17237 Athens, Greece; dkoutsou@phed.uoa.gr

In the original publication [1], there was a mistake in Table 2 and Table 3 as published. We corrected some data in these two tables because the raw data were modified to standardized and analogy index data. The corrected Table 2 and Table 3 appear below. We state that the scientific conclusions are unaffected. This correction was approved by the Academic Editor. The original publication has also been updated.

## Figures and Tables

**Table 2:** Means and SDs of Standard score differences for DCD and non-DCD Groups in motor skills.

Mοtοr Variables	DCD		Non-DCD	
	M	SD	M	SD
Manual dexterity 1	1.65	1.29	0.38	0.7
Manual dexterity 2	3.36	1.24	1.06	1.25
Manual dexterity 3	2.53	1.92	0.53	0.73
Aiming and Catching 1	2.7	1.8	0.5	0.68
Aiming and Catching 2	2.73	1.4	0.47	0.86
Static Balance	2.2	1.57	0.48	0.79
Dynamic Balance 1	2.56	2.3	0.73	1.25
Dynamic Balance 2	2.46	1.67	0.75	1.2
Total MABC score	20.19	7.67	4.9	2.96

**Table 3:** Means and SDs of Standard score differences for DCD and non-DCD Groups in Planning Scale and Cognitive abilities.

Cognitive Variables	DCD		Non-DCD	
	M	SD	M	SD
Matching Numbers 1 (0–151 s)	130.3	33.1	85.5	32.42
Matching Numbers 1 (corrects)	6.76	1.57	7.9	0.57
Matching Numbers 2 (0–151 s)	150.3	3.65	148.6	13.14
Matching Numbers 2 (corrects)	3.03	1.60	4.66	1.26
Planned Codes 1 and 2 (0–121 s)	121.0	0.0	121.0	0.0
Planned Codes 1 (corrects)	10.8	6.18	19.5	7.86
Planned Codes 2 (corrects)	9.56	3.82	15.16	5.34
Planned Connections	5.67	3.75	10.13	2.5
Planning standard scaled total score	82.86	14.5	104.9	13.5
